# Procidentia Uteri and Its Treatment

**Published:** 1878-12

**Authors:** Henry P. Wenzel

**Affiliations:** Theresa, Wis.


### Selections.

### PROCIDENTIA UTERI AND ITS TREATMENT.

By HENRY P. WENZEL, M.D., Theresa, Wis.

There is no other disease in the domain of gynæcology that produces so much discomfort and suffering as procidentia uteri. Whether it be caused by brutal treatment during labor, or falling or jumping, or from atonicity of the uterine ligaments or the walls of the vagina, or the displacement resulting from pressure—there is but one way to correct the malposition: reduction of the organ to its normal position and retention by proper appliances.

It is useless, here, to detail the anatomy or the relations of the parts involved. The redundancy of the vaginal walls and debility of the uterine ligaments are prime causes in this lesion. So soon as the womb is forced below its normal site for any length of time, congestion and, later, hypertrophy, result; the increased weight of the organ further displaces it, and with the prolapsed uterus follow the ovaries, bladder and rectum. During the first stage of prolapsus—really only a descent—there may be no inconvenience, nor erosions of the mucous membrane; but when the os tincæ presents at the vulval fissure, or the greater part or the whole uterus hangs pendant between the thighs, the mucous tissues are rapidly ulcerated or totally destroyed. In recent cases the uterus is congested and eroded or fissured, but later it is covered with erosions, ulcers and scabs; a fetid, thin, sanious pus exudes, which may endanger the patient's life by absorption or inhalation of septic matter. The bladder and rectum are displaced, the latter forward and downward, the former downward; the lower segment of the uterus is partly everted; the cervical canal forcibly dilated; the vaginal walls inverted, and the pelvic vault weakened; the intestines sink into the pelvic cavity and produce an unnecessary pressure on the displaced organs. The excruciating agony, the rectal and vesical tenesmus, and the host of concomitant symptoms, must necessarily result as a consequence of the malposition of all the parts involved. Is it a wonder, then, that the unfortunate patient prays for death to end her misery? Yet this lesion is generally easy to correct, and taken in time may not only be relieved, but cured in young cases. There are legions of appliances to correct the trouble, but how many deserve notice or a name? How many of them relieve only temporarily, or, if you please, increase the evil? How often is a pessary unnecessarily applied, and by its pressure does infinite harm?

Why do these cases pass from one hand to another? Each one attempts to relieve; he who "quacks" the case longest gains the largest fee, and the disgusted patient seeks relief elsewhere. The difficulty lies here: the natural laws involved in this lesion are disregarded; the malposed anatomical structures ignored; no attention is paid to their relations, nor to physiological functions; good common sense is of more value than theoretical speculation. Besides, there are many pretenders, claiming to be gynæcologists, who could not differentiate a prolapsed rectum from the prolapsed womb, or between the latter and prolapsed vagina; consequently, the treatment must be worse than useless. When an accurate diagnosis is made, the appliance to be worn for the correction of the deformity should be carefully selected and carefully adjusted, that it may fit accurately. It is as essential for a pessary to fit accurately as for a boot to fit the foot nicely. Pressure must, under all circumstances, be avoided. Dangerous and fatal inflammation may result from unnecessary pressure at any time.

Sometimes a mechanical appliance is altogether out of place. In general debility of the system, and atonicity or relaxation of the stays of the uterus, a simple tampon, locally, and the bitter tonics and iron internally, are followed by better results than the best pessary ever invented.

Three years ago a lady, aged fifty-three, called to be treated. She could scarcely be out of bed. She suffered from general exhaustion, nervousness, etc. A year previously her health failed, and she had prolapse of the womb. A Hodge closed-lever pessary had been introduced by a physician, but her condition did not improve. She began to complain of pain and tenderness in the pelvis, which continued unremittingly, and was of course attributable to the pessary. A thin, fetid liquid discharged constantly in small quantities from the vagina, and rigors, followed by flushes of heat, supervened. This was also laid to the presence of the pessary, and the attending medical adviser directed that the pessary must remain to cover his careless blunder and hide the damage done as long as possible. A vaginal examination revealed the pessary imbedded in the tissues. It was removed, and a gush of fetid, sanguinolent fluid followed. The vaginal walls were deeply ulcerated, and the posterior part of the cervix was nearly destroyed by the large sores in its substance. Locally, she was treated by a tampon, saturated with carbolized glycerine, and internally the bitter tonics and iron. In two months she performed all her household duties without any mechanical support whatever; nor has she worn any appliance whatever since; yet at the present time she is in good health, and there is no procidentia or falling of the womb. The treatment was simple and rational, and the original trouble being removed, all danger of prolapse had vanished.

There are cases where ligaments holding the uterus *in situ* have been overstretched and exhausted; degenerative changes have taken place. Here complete recovery is impossible. The organ can be replaced, and must be kept in proper position by accurately fitting mechanical appliance, to remove the difficulty and relieve the patient's distress. The best instrument for the purpose I have found to be that manufactured by Dr. S. S. Staufer, of Philadelphia. It is composed of two parts, namely, an abdominal belt of strong elastic rubber, and a hard rubber stem cup, resting on two soft rubber tubes which pass through its base, and are attached to the belt in front and behind by buckles. The cup is placed on a curved stem to fit the pelvis, thereby avoiding useless friction. The cups are of different size and shape. The instrument is easily applied, removed and cleaned. It cannot get out of order. There is no caustic action, for gold and silver may corrode—rubber never. The instrument may remain *in situ* during urination and defecation, and does not cause annoyance in the sitting or reclining posture. All instruments must be accurately fitted, and Dr. Staufer gives exchange privileges until correct.

The two following cases were recently treated for procidetia uteri. One is a young mother of twenty-four, in excellent health, and the other an old lady, about seventy-two years of age, suffering from bronchitis.

Case 1.—Mrs. W. jumped from a lumber wagon in October, 1877. She was in her third month of pregnancy. There was sudden pain and distress, and the attending physician found a prolapse of the second degree, reduced the dislocation, and applied a ring pessary which came away a few days after (application). She was delivered, at term, of a large, living, female infant. The midwife allowed her to get up on the third day. She felt immediate pain, tenderness and inconvenience, which constantly grew worse. I saw the case for the first time two weeks later, and found complete procidentia. The uterus was as large as a fetal head, dusky red, and had some fissures on the exterior. The vagina was pulled down and inverted; there was internal vesical and rectal tenesmus, intense pain in the knees, etc. The organ was with difficulty replaced, and a tampon saturated with carbolized glycerine placed against the os. The vagina was packed with cotton. The dressings were changed every two days. She was kept supine for two weeks. There was improvement from the first, pain and tenesmus ceased, and appetite and sleep returned. She received no medicine internally.

Four weeks later the uterus was much diminished in size and free from ulcerations. I fitted one of Staufer's stem-cup supporters (cup 2½ inches). She performed her household duties at once with ease; the uterus was perfectly retained. She bound and carried sheaves during harvest, and does all kinds of farm work besides her household duties, and is happy. The instrument is worn since the 1st of May, and the relief is permanent.

Case 2.—Mrs. F., aged about 72, was injured by an ignorant midwife, at the birth of her last child. She was treated with variable success by many physicians, and wore nearly every variety of pessary in the market without any benefit. Some called her trouble "prolapse" of the bladder; one "prolapse" of the vagina; one "prolapse" of the rectum. She was constitutionally treated, without any benefit whatever.

*Status præsens*, July 31st, 1878. Saw her the first time to-day. She is broken down in health; has *facies uterina;* eyes very weak; violent paroxysmal headache; anorexia and insomnia; locomotion is impossible; there is violent pain in both knees and in back; her pulse is neither slow nor fast, but feeble; she suffers from chills and flushes of heat, constant nausea and frequent vomiting. There is vesical and rectal tenesmus and an acrid diarrhœa. Her person is very offensive. There is also chronic bronchitis.

The uterus is completely prolapsed, and lies between the thighs. The mucous membrane is destroyed and the surface full of ulcers, which are covered with scabs and crusts; the ulcers extend to the muscular tissue, and exude a thin, fetid, sanio-pus. The external os is widely dilated and cervix partly everted; the cervical canal is a mass of ulcerated tissue; the fundus is also implicated; cavity nine inches deep. The vaginal walls are inverted and full of fetid ulcers. The bladder is drawn downward and the rectum downward and forward, forming a pouch, which is filled with fecal matter. In the upright position the intestines sink into the pelvic cavity.

The entire diseased mass was bathed with solution of thymol, which removed the fetor at once and permanently. The organ was with difficulty replaced, and a tampon saturated with solution of thymol—thymol, 1 gm; glycerine, 120 gm.; water, 600 gm., applied. This forms a clear solution, and has a specific gravity of 1.026. It may be further reduced if found to "burn" too much when applied—placed against the cervix uteri. The vagina was moderately packed with cotton. For two weeks the dressings were renewed night and morning, subsequently every three or four days. Internally, pulverized glycyrrhizæ

comp., of the Prussian Pharmacopœia, a heaped teaspoonful at night, in a tumbler of water, during her time of treatment.

August 1st. Less nausea, and headache less violent; tenesmus only moderately severe. *There is no fetor;* appetite poor; local treatment same; internally (Wyeth's) elix. cinch. pepsin and bismuth. August 3d. Appetite improved; no pain whatever on walking; œdema of uterus subsiding; ulcers covered with granulations; uterine cavity six inches; treatment same. August 8th. Uterine cavity four and a half inches; healthy granulations. Internally four drops fl. ext. æsculus glabera thrice daily. August 23d. Uterine cavity three inches; uterine walls flaccid; no pain; no œdema; ulcers healed; general health much improved; treatment continued. August 28th. Applied a Staufer supporter, with two-inch oblique stem-cup; all other treatment stopped. September 4th. Took a ride of twenty-four miles in a carriage yesterday, without inconvenience. General health very good. September 30th. Uterus held perfectly in position. There is neither discomfort nor annoyance. At the present time her general health is good; she attends to her few household duties, and the instrument has relieved the trouble permanently.

In conclusion, I wish to call attention to the use of thymol solution in uterine diseases. An extensive trial during the last six months, in diseases of the womb, convinces me that it is a valuable adjunct in uterine therapeutics, and superior to carbolic acid.

1st. It cannot be made strong enough to act as a cauterant; thymol in substance feebly cauterizes.

2d. Thymol solution destroys the fetor instantly and effectually.

3d. Thymol solution leaves the grateful odor of *thymus vulgaris.*

4th. Thymol solution heals the ulcer more rapidly.

5th. There is no danger of toxic symptoms following the local application of thymol solution; it may be taken internally more freely than carbolic acid, without producing dangerous consequences.—*Med. and Surg. Reporter.*

